# A qualitative study—Patient experience of tactile massage after stroke

**DOI:** 10.1002/nop2.515

**Published:** 2020-05-29

**Authors:** Berit Seiger Cronfalk, Elisabet Åkesson, Jill Nygren, Anita Nyström, Anna‐My Strandell, Jorge Ruas, Mia von Euler

**Affiliations:** ^1^ Department of Nursing Red Cross University College Stockholm Sweden; ^2^ Division of Nursing Department of Neurobiology, Care Sciences and Society Karolinska Institutet Stockholm Sweden; ^3^ Division of Neurogeriatrics Department of Neurobiology, Care Sciences and Society Center for Alzheimer Research Karolinska Institutet Stockholm Sweden; ^4^ R & D Unit and Rehabilitation and Primary Care Stockholms Sjukhem Foundation Stockholm Sweden; ^5^ Department of Physiology and Pharmacology Molecular & Cellular Exercise Physiology Karolinska Institutet Stockholm Sweden; ^6^ Department of Clinical Science and Education Karolinska Institutet Stockholm Sweden; ^7^ Department of Medicine Södersjukhuset Karolinska Institutet Stockholm Sweden; ^8^ Department of Clinical Pharmacology Karolinska University Hospital Stockholm Sweden

**Keywords:** nurses, nursing, RCT, skin massage

## Abstract

**Aim:**

The aim was to evaluate emotional experiences of gentle skin massage, combined with regular rehabilitation in patients shortly after being diagnosed with stroke.

**Design:**

A randomized study with two groups: standard individualized rehabilitation and tactile massage for 20 min three times per week (max nine times) or individual standardized rehabilitations.

**Methods:**

This study applied a qualitative approach using semi‐structured questions to evaluate experiences of receiving tactile massage among patients with first‐time‐ever stroke. The interviews lasted between 6–25 min and analysed using manifest content analysis. Data was collected between 2015‐2017. This study applies to the COREQ checklist.

**Results:**

Eight patients >18 years of age participated. The participants experienced emotional worries especially during the night hours affecting their sleep negatively. Receiving tactile massage was reported to relax and to ease worries and anxiety momentarily, during the session and for a longer period. The results also show that physical touch generates feelings of closeness. The findings will be presented in two categories: *Human touch* and *The future*.

## 1 INTRODUCTION

Stroke is a common disease and almost 17 million persons suffer stroke yearly in the world, making it the second most common cause of death and the third leading cause of disability‐adjusted life‐years lost worldwide. Having a stroke is for most a deeply frightening experience and aiming to regain lost function is often challenging on many levels (Hankey, 2017).

## 2 BACKGROUND

Among symptoms following stroke pain and anxiety are described as affecting the patient's quality of life and is greatly underdiagnosed and undertreated (Campbell Burton et al., 2013; Harrison & Field, 2015). According to Harrison and Field (2015), clinicians need to better identify those at risk of pain following stroke by analysing types of pain as well as options of treatments. Regarding anxiety disorders during the first weeks after stroke approximately one third has been described to suffer from the symptom (Rafsten, Danielsson, & Sunnerhagen, 2018). Ayerbe, Ayis, Crichton, Wolfe, and Rudd (2014) report that more than one third of patients showed signs of anxiety affecting their well‐being and quality of life negatively for as long as 10 years after their stroke.

Tactile massage (TM), light touch and pressure following an established instruction, has been shown to increase quality of life and well‐being over a range of conditions such as acute coronary syndrome, rheumatic disease, spinal cord injury, diabetes, stroke, dementia as well as for patients subject to palliative care (Alimohammad, Ghasemi, Shahriar, Morteza, & Arsalan, 2018; Bergsten, Petersson, & Arvidsson, 2005; Chase, Jha, Brooks, & Allshouse, 2013; Cronfalk, Strang, Ternestedt, & Friedrichsen, 2009; Pedersen & Bjorkhem‐Bergman, 2018; Wandell et al., 2013). Previous studies on TM after stroke, have to our knowledge, mainly focused on activities of daily living (ADL) and quality of life (QoL). TM is described to decrease anxiety levels in some of these patients as well as reducing pain in patients with cancer (Cooke, Emery, Brimelow, & Wollin, 2016; Myers, Walton, & Small, 2008; Pedersen & Bjorkhem‐Bergman, 2018). Thus, we hypothesized that stroke patients could potentially benefit from TM. Smaller studies on TM and stroke have been performed and reported in national journals. However, studies on patient experience of TM in stroke are lacking in the international literature. Therefore, we interviewed patients participating in a randomized controlled trial comparing the addition of TM (as an active comparator) to regular rehabilitation in an inpatient specialized neurological rehabilitation clinic and compared with ordinary rehabilitation alone. The aim of the study was to evaluate emotional experiences of gentle skin massage, TM, combined with regular rehabilitation in patients shortly after being diagnosed with stroke.

### 2.1 Design and methods

To enhance the quality and transparency of this study, the COREQ checklist was applied.

This qualitative interview study used convenient sampling and including eight patients. Patients admitted to a specialized neurological rehabilitation clinic meeting the inclusion criteria and giving their informed consent were randomized to either (a) regular rehabilitation + TM or (b) regular rehabilitation alone. Analysis was performed using manifest content analysis (Graneheim & Lundman, 2004).

Convenience sampling was applied including four participants in each group (TM and control). Inclusion criterion: adults ≥65 years of age with first‐time‐ever stroke, admitted to one rehabilitation unit, within the time frame of 30 days after stroke, able to understand and communicate verbal and written Swedish. Exclusion criteria: patients with MOCA ≤ 16 (Montreal Cognitive Assessment (MoCA) version 7.0; Nasreddine et al., 2005) and/or with severe aphasia affecting their ability to give informed consent and to participate. The study was conducted at one specialized inpatient rehabilitation unit with 23 beds. Patients in need of rehabilitation due to neurological disorders, including stroke were admitted to the ward. Patients with stroke were admitted directly from the acute stroke care units in Stockholm. The length of stay for patients in stroke rehabilitation was 17–21 days in the ward followed by referral to primary care rehabilitation by regional neuro‐teams. Each patient together with the rehab team (including physiotherapist, occupational therapist, physician, Registered Nurse (R.N.), speech therapist, neuropsychologist, nutritionist, social worker) designed a rehabilitation plan according to the individual needs. Nursing staff and physicians were available at all hours while the rest of the rehab team was available 5–6 days per week.

The clinical research team included one physician and one RN both with extensive experience of research and intervention studies, along with one additional RN and two assistant nurses. The study included training sessions with the TM expert in hand and foot massage prior to the patient inclusion to confirm intra‐ and inter‐person reproducibility of the TM routine given by the team members. The extent of the training was 4 × 4 hr including a theoretical part describing touch and its neurophysiological effect followed by practical training sessions giving/receiving hand‐ and foot massage. All staff were informed about the study prior to its start, at regular staff meetings. For inclusion to be successful, it was important to secure that staff were informed about the study and understood the study protocol. Ward supervisors and senior physicians were informed separately as they were involved in the daily inclusion process with the research team.

To secure that the TM addition and study protocol could be carried out for all study participants without interfering with the daily schedule of training sessions, the research team conducted the study during afternoon and evening hours (2.30 p.m.–9 p.m.), except for Fridays (11 a.m.–7 p.m.). A written structured manual guided the research team through the study protocol and working shift. During the afternoon study, staff identified new admissions, gave verbal and written information and collected written consents. If a patient gave informed and written consent to participate, he/she was randomized to one of the two groups by a blind lottery approach conducted by the research team. The patients were then informed about what group they should attend (one patient withdrew his/her participation). This procedure was followed by the study staff handing out baseline questionnaires. A time schedule was decided and later, after the evening meal the participants were introduced to intervention or rehabilitation alone. Questions about their sleep were registered before and after each TM session and at equivalent time points also in the rehabilitation alone group. This was repeated three times a week (Mon‐Wed‐Fri) for 3 weeks (nine sessions).

Group 1. TM and regular rehabilitation. Participants in this group followed their schedule of daily rehabilitation (individual and group rehab training) and in addition chose between hand or foot massage (20 min). Light touch massage was applied employing slow strokes and light pressure using structured movements. Light scented vegetable body oil (Pomegranate Regenerating Body oil and Sea Buckthorn Body oil, Weleda^©^) was used to minimize skin friction. The participants received the TM lying down in their own hospital bed. During the massage study, staff were informed not to initiate a conversation but answered questions if posed during the massage session.

Group 2. Regular rehabilitation alone. Participants in this group followed their schedule of daily rehabilitation (individual and group rehab training) alone without any additional intervention.

Minimal Insomnia Symptom Scale (MISS) (Broman, Smedje, Mallon, & Hetta, 2008), blood pressure and heart rate were registered prior to and after the TM intervention, while only once after resting for 20 min in the control group and will be presented in a separate report.

### 2.2 Analysis

Individual tape‐recorded interviews were conducted at a time convenient for the patient on the last day of being included in the study. Semi‐structured questions were posed: “Can you describe how you feel today?” this was followed by; “What made you want to participate in this research study?” “What are your experiences from receiving massage?” “What did you think when you weren't randomized to the massage group?” as well as follow‐up questions. The interviews lasted between 6–25 min and were conducted by a senior researcher. The interviews were transcribed by a professional transcriber not involved otherwise in the study and analysed using Graneheim and Lundman (2004) approach to manifest content analysis. The analysis was conducted by the authors until consensus was reached.

### 2.3 Ethics

Patients included in the study received verbal as well as written information about the study at the time of admission to the ward. The persons that gave their informed consent received a copy of the written consent along with telephone numbers and e‐mail addresses to the research team. All were informed that they could at any time and without further explanation withdraw from participating in the study without affecting the regular rehabilitation care. The study was approved by the Ethical Review Board in Stockholm, Sweden (reference number: Dnr 2015/1627‐31).

## 3 RESULTS

Eight patients over the age of 65 (five women and three men) at one stroke rehabilitation unit participated in the study. The main results show that participants in both groups experienced emotional worries especially during the night hours mostly affecting their sleep negatively. Participants in the massage group did express that the massage was helpful to relax and to ease worries and anxiety momentarily, during the massage session and in some cases for a longer period of time. The aim of this study was also to evaluate other aspects of receiving massage, here described as closeness from a person other than family members.

The results show that the participants wished to participate in the study as they had hoped to be selected to the TM group. Their pre‐conceived notion of massage was for some from previous experiences, while others expressed an understanding of it being nice and adding value to life. Another reason for choosing to participate was curiosity and the possibility to contribute to research in stroke rehabilitation:

I found it enjoyable, but at first I got worried that they would need to take a lot of blood samples, but they did not (women 1 TM)

Well, If I had been selected for massage, I would have been happy, but I wasn't (male 2 Control group)

The findings will be presented in two main categories: *Human touch* and *The future*.

### 3.1 Human touch

This category will be described in two subcategories: *The importance of touch* and *Inner worries*. The importance of touch is emphasized as well as a need for touch but also its implications for patients in rehabilitation after stroke.

### 3.2 The importance of touch

Human touch in general was described as important and included different dimensions by the participants in the massage group. The predominating experiences were feelings of comfort, calmness and a sense of respite. The experience of massage was also described as intense and generating peacefulness. Another aspect brought forward by the participants was the dimension of human touch in relation to feeling lonely. Touch was described as a resource that varied in intensity depending on one's life situation, with or without family members and close friends. Even though living in a tightly knit family the experience of touching each other was for one person described as brief and temporary:

I believe that many patients that live alone, experience touch of another human being as rare, a rare thing. I, myself have grandchildren and get hugs from them and from my partner (for 60 years). I don't have the same need. Still, their touch is short and not so intense. The massage is quite intense for twenty minutes. In that sense it is a great difference. The other touch (from family members) is in a sense shallower you know, kisses and hugs. It is not comparable (male 1 TM)

The contact between the massage therapist and the participant was also described as a positive experience allowing feelings and moments of stillness:

Here I am lying on my bed being touched by another person, who gives a warm and kind impression. It is lovely and I am thinking ‐ I feel relaxed – and distracted in my thoughts and worries and even anxiety (male 1 TM)

As I am overwhelmingly satisfied, I can only say that nothing I have previously experienced have had the same effect. I felt relaxed. I think it must be the same for everyone, I mean the deep sense of relaxation that the massage contributes with (women 2 TM)

One patient in the control group did not agree to be tape record, therefore a written statement was undertaken.

I have previous experience from working as a beautician and with complementary therapies and am well aware of the importance of touch. I was disappointed when I was not picked for the massage group (women 2 Control group)

### 3.3 Inner worries

Shared by all, participants described their feelings of worry or anxiety and that it affected their night sleep. The participants' perception was that most fellow patients sensed the same feelings:

Most patients here are tense and worried. And I know as there are many that need to take a pill to be able to sleep, even if I myself haven't yet (women 1 Control group)

The massage was described as a possible resource to ease their worries, and even in the control group of participants not receiving massage, it was thought to contribute positively.

I did not get massage myself, but I have heard from others that it is calming and peaceful (male 2 TM)

For those who received massage, it was described as a respite from worries and anxiety. It was illustrated vividly as exemplified by the following quotations:

It was a way to disengage from worrying thoughts and anxiety and so on. And that's what was important. One could for example be scratched on ones back instead, but it was the contact. To feel the warm hand touching me, that was actually peaceful. Very comforting, I could dissolve my thoughts from my brain, just disappear and not think about all the strange thoughts and of being scared, just disappear into the massage (male 2 TM)

The emotional experiences of gentle massage and human touch were described as unexpected. It was described as a break from their otherwise intense and active daily schedule of stroke rehabilitation. The quotation below will give voice to one participant experience:

I have never thought about getting massage just on my hands and that it could have such a large and deep impact on my emotions as well as everything else, it is fantastic (male 1 TM)

### 3.4 The future

In this category, the participants described their thoughts about the future life following stroke and how it would affect their time ahead. Different aspects of worrying for the future were expressed. For some, the lack of information about what to expect concerning their own health, how and if it would affect their families and if there is a component of heredity associated with stroke. It was considered troublesome, but also information about how they would cope in everyday life was important:

This stay has helped me a lot (male 2 Control group)

I have been thinking a lot, dwelled since it happened as no one says anything. And then I heard that two other patients on the ward were here for the second time after new strokes, Ouch (male 1 TM)

My daughter calls me every single evening asking, ‐ Mum, what risk is it that you will have another one? and I don't know (women 2 TM)

Fear of the future was voiced by all. Fearing the unknown and for what the future might hold were thoughts mainly during the night hours. Their experiences of sleepless nights and worries were described by most:

I haven't slept a single whole night since my stroke. It's worrying, I wake up and can't go back to sleep (male 1 Control group)

How can I feel calm after a thing like this? It's impossible, especially as I see other patients not being able to speak anymore… (male 1 Control group)

However, some described no immediate worries about the future nor sleepless nights. This was mainly participants in the control group. Even though life was experienced to fluctuate, it was in general considered to be positive:

I don't know how I feel, it's ups and downs from one moment to the next (women 2 TM)

I usually have no problem sleeping, I wake up and go to the bathroom and then go back to sleep again (women 2 TM)

## 4 DISCUSSION

The results show that being considered for and taking part in a research study was experienced as positive by the patients even though they were newly diagnosed with a first ever stroke. The research team had no previous experience of introducing tactile massage to this group of patients and was therefore positively surprised by the patients' willingness to participate in the study. Taking part in the study was described as gaining positive attention, generating feelings of being special. This agrees with Riet, Dedkhard, and Srithong (2012) who described the importance of listening to patients' own narratives regarding feelings and experiences during their rehabilitation process as means of the convalescence process (van der Riet et al., 2012b). It became clear that all participating patients had needs to narrate and to tell “their story” about how they experienced and handled their illness.

Patients in the massage group described human touch and the closeness and warmth of another person as important. This included feelings such as being described by van der Riet et al. (2012b) and here uttered as generating comfort, calmness, respite, intensity and peacefulness. In neurophysiological research, Bjornsdotter, Morrison, and Olausson (2010) and Loken, Wessberg, Morrison, McGlone, and Olausson (2009) could identify specific touch receptors in hairy skin, C‐tactile nerves associated directly to parts of the brain connected to feelings (Bjornsdotter et al., 2010; Loken et al., 2009). Their studies show that slow and gentle touch and stroking of the skin activates the C‐tactile nerve fibres facilitating emotions of well‐being (Morrison, Loken, & Olausson, 2010). Furthermore, it is known that gentle skin touch releases oxytocin with known positive effects on muscle tension, pain and relaxation (Ellingsen et al., 2014; Morrison et al., 2010).

The massage offered a deeper emotional dimension than what the patients had expected. For some, daily touch of another person had previously not always been present, as they lived alone. Yet, patients that was part of a family did not always consider touching to be present either, depending on their personal situation. In some cases, the patients described touching a family member as both brief and temporary. The experience of how it felt to receive gentle tactile massage was therefore an important insight into what touch could implicate. In rehabilitation, the multi‐professional team of staff play a pivotal role. van der Riet, Dedkhard, and Srithong (2012a) emphasize that nurses should use a holistic approach when caring for patients with stroke in their recovery. This is in line with Hankey who suggests that experiencing stroke, indeed is related to suffering and as such a frightening experience (Hankey, 2017). This is also in agreement with other studies suggesting that patients suffering from stroke experience complex feelings of anxiety regarding their disabilities and future consequences (Alimohammad et al., 2018; Harrison & Field, 2015; van der Riet et al., 2012b). Alimohammad et al. (2018) showed changes in patient anxiety following sessions of hand or foot massage after stroke, suggesting that massage as means of relaxation could be helpful to ease the patients suffering. This is in line with Golding et al. (Golding, Fife‐Schaw, & Kneebone, 2017, 2018; Golding, Kneebone, & Fife‐Schaw, 2016) and their studies, as they introduced relaxation techniques by a self‐help programme. The results show positive effects as it reduced anxiety in stroke survivors, both short and long term.

This is comparable with our results, as the patients experienced anxiety regarding their own health and outlook during their rehabilitation. For most, but not all, it had a negative effect on their night sleep. Patients received medication to sleep, but later at night woke up and found it difficult, fearful and hopeless to go back to sleep again and thereby becoming a problem. However, during the massage sessions, the patients described how they could relax and be at peace.

Although patients in our control group did not receive massage, they were able to express appreciation for the other patients' experiences. Some patients, all in the control group, did not talk about worries for the future or sleeping problems. In a previous review of sleeping difficulties, the prevalence of difficulty falling asleep was found to vary between 8%–48% and difficulty staying asleep to be slightly higher, between 19%–65% (Baylor, Yorkston, Jensen, Truitt, & Molton, 2014). Another recent review found anxiety to be present in one third of patients during the first 2 weeks after stroke (Rafsten et al., 2018). Our study addressed this topic using a qualitative approach trying to better comprehend the complex emotional situation patients with first ever stroke find themselves in. We have shown that a short session of gentle hand or foot massage had a positive effect as it reduced patients' experience of anxiety and worry and increased well‐ being. The effect needs however to be explored further in a larger sample.

## 5 CONCLUSION

The objective of this qualitative study was to evaluate emotional experiences of gentle skin massage among patients with first ever stroke while in active rehabilitation. To improve the understanding, multi‐professional clinical teams a‐ long‐ side research teams need to evaluate how and what is relevant as support strategies in clinical rehabilitation. An ethical consideration of importance to consider when including newly diagnosed patients with stroke is their willingness to participate in research.

## 6 LIMITATIONS

This qualitative study has limitations concerning the low number of patients participating. Therefore, the research team decided to limit the presentation of demographic data as there was an imminent risk of the patients' identities being recognized. The limitation to take into consideration must therefore be the inclusion criteria. With a broader nationwide inclusion, the risk of identifying individuals would decrease considerably. Even if this sample is relatively small and the interviews varied in time, most participants contributed richly.

## CONFLICT OF INTEREST

The authors declare no conflict of interest.

## AUTHORS CONTRIBUTIONS

BSC, EÅ, ME: Substantial contributions to the conception and design of the study. BSC, EÅ, AMS, AN, JN: Data collection. BSC: Analysis of data. BSC, ME: Drafting the work. BSC, ME, EÅ, JR, AMS, AN: Manuscript writing and approval of the version to be published. BSC, EÅ, ME: Accountable for all aspects of the work.
